# Performance of seven criteria to assess CA125 increments among ovarian cancer patients monitored during first-line chemotherapy and the post-therapy follow-up period

**DOI:** 10.4155/fsoa-2017-0023

**Published:** 2017-07-18

**Authors:** Suher O Abu Hassan, Dorte L Nielsen, Malgorzata K Tuxen, Per H Petersen, György Sölétormos

**Affiliations:** 1 Department of Clinical Biochemistry, Nordsjællands Hospital, University of Copenhagen, Hillerød, Denmark; 2 Department of Clinical Research, Nordsjællands Hospital, University of Copenhagen, Hillerød, Denmark; 3 Department of Oncology, Herlev & Gentofte Hospital, University of Copenhagen, Herlev, Denmark; 4 Norwegian Quality Improvement Primary Care Laboratories (NOKLUS), Section for General Practice, University of Bergen, Bergen, Norway

**Keywords:** assessment CA125 criteria, CA125, CA125 increments, clinical progression, lead time, monitoring, ovarian cancer

## Abstract

**Aim:**

To investigate seven CA125 criteria to monitor progressive ovarian cancer among patients with stage IC–IV disease.

**Materials & methods:**

Four criteria were used to asses CA125 increments starting from concentrations ≥35 U/ml and three criteria to asses increments starting from concentrations <35 U/ml.

**Results:**

A total of 231 patients were allocated to CA125 monitoring. The performances of the CA125 criteria were similar with sensitivities of 30–55%, negative predictive values of 28–46%, positive predictive values of 90–100% and median lead times of 26–87 days.

**Conclusion:**

The criteria showed low sensitivity and inability to exclude progressive ovarian cancer. The study suggests that CA125 information cannot stand alone but should be considered used in conjunction with other investigative procedures.

## Lay abstract

Few have validated the CA125 progression criteria proposed by Rustin *et al*. in 1996 and in 2001. These two criteria were officially recommended by the Gynecological Cancer Intergroup to be incorporated into clinical trials in 2010. Among other criteria, we validated the Gynecological Cancer Intergroup CA125 progression criteria in an ovarian cancer population regarding sensitivity, specificity, positive predictive value and negative predictive value. We found that the criteria had low sensitivity and ability to exclude tumor growth. We believe that the clinical utility of CA125 may be questioned, and there is a need for supplementary markers and alternative assessment criteria.

A change of tumor size is routinely measured by radiological imaging according to the Response Evaluation Criteria in Solid Tumors (RECIST 1.1) [1]. However, this may be difficult among patients with ovarian cancer as they often have no macroscopic detectable disease after initial surgery or they present with widespread diffuse peritoneal metastases [2–4]. The serological tumor marker, cancer antigen (CA125), is frequently added as a biochemical monitor of patients with epithelial ovarian/fallopian tube or primary serous peritoneal cancer [5]. However, it is a challenge to define increments of CA125 concentrations that reliably correlate with increasing tumor burden, in other words, recurrence and progressive disease. In the last three decades, a number of evaluation criteria have been proposed to interpret serially increasing CA125 concentrations [5–20].

Recently, a systematic review [21] identified seven criteria to assess CA125 increments from below to above the applied cut-off concentration and from above the applied cut-off to higher levels as proposed by Rustin *et al*. [6,8] and by Tuxen *et al*. [7,20]. The criteria by Rustin *et al*. were generated from epithelial ovarian cancer (EOC) patients during follow-up after first-line chemotherapy and incorporated into the RECIST 1.1 by the Gynecological Cancer Intergroup [1]. The criteria suggested by Tuxen *et al*. were generated from ovarian cancer patients monitored during first-line chemotherapy as well as the subsequent control period [7,20]. The seven criteria identified in the review were further compared in a Phase I monitoring trial according to the design recommendations from the European Group on Tumor Markers (EGTM) [22]. They were compared under standardized conditions, and their individual ability to detect early tumor growth was evaluated in a preclinical model system based on computer simulated CA125 concentrations [23].

The current Phase II monitoring study estimated whether the criteria that performed the best [24] in the simulation model also performed the best when applied to serial CA125 concentrations obtained from ovarian cancer patients monitored during first-line chemotherapy and the postchemotherapy follow-up period [23]. The study also estimated whether the criteria introduced by Rustin *et al*. were useful during first-line chemotherapy even though their criteria were generated from patients during follow-up after first-line chemotherapy. Overall, the current study was performed to challenge a previous report by Rustin *et al*. which suggested that CA125 should not be used as a standard test for monitoring patients with ovarian cancer.

## Materials & methods

### Design

The study complied with the general recommendations for study design as specified by the Standards for Reporting Diagnostic Accuracy Studies (STARD) [25]. However, it was not a cross-sectional diagnostic study but a longitudinal monitoring trial based on serial measurements of CA125 among individual patients. Additionally, the study followed a phased approach as proposed by the EGTM and was designed as a prospective Phase II biomarker monitoring trial embedded into clinical drug trials where the tumor marker investigation was secondary to the clinical drug trial [22,26]. Accordingly, the clinical information and the results of the imaging analysis were available to the staff at the ward, and so were the CA125 concentrations. Phase II monitoring trials validate the performance of the investigated biomarker in patient cohorts by applying assessment criteria for marker progression that were identified as promising by previous preclinical Phase I trials. Phase II monitoring trials estimate the ability of the biomarker to identify, exclude and predict a change in disease status.

### Patients

Patients with newly diagnosed, histologically verified EOC with International Federation of Gynecology and Obstetrics (FIGO) stages IC–IV were included. The study was conducted at the Departments of Oncology, Herlev and Gentofte Hospital as well as Nordsjællands Hospital during 1995–2001. Date of primary surgery, the beginning and the end of first-line chemotherapy as well as follow-up were registered for all patients. Evaluation of the disease by gynecological examination, ultrasound and/or computer tomography was repeated at every third treatment cycle, and every 3 months during the first 3 years of follow-up and every 6 months thereafter until a total surveillance period of 5 years. Additional evaluations were performed when clinically indicated. The clinical status in terms of progressive disease was recorded according to the WHO criteria because these were in use at the time of the clinical evaluations [27]. The clinical evaluations according to the WHO criteria were the reference against which the results of the CA125 evaluations were compared. The study was approved by the Regional Ethical Committees (KA 94162m) (H–3–2013–FSP43) and The Danish Data Protection Agency (1995–1200–655), (2013–41–2366). The databank with the cumulated clinical and CA125 data was assessed in 2015.

### Collection of blood samples

Samples were collected on the days of treatment and the days of clinical evaluation. Additionally, if possible, samples were collected when routine analytes were requested outside the scheduled time points.

### CA125 measurements

Concentrations in serum were measured prospectively a few days after sample collection with the ELISA–CA125 II assay, a solid-phase two-site immune radiometric assay, from CIS Bio International. Following August 1996, the CA125 concentrations were measured with the Immuno 1 CA125 II assay, a one-step solid phase enzyme immunoassay, from Bayer. The reference interval (the 95 percentile value) used for both assays was <35 U/ml as recommended by Bast *et al*. [5].

### Quality assurance

To ensure a stable analytical quality throughout the study, three control samples were included in each assay run having different concentrations of the analytes. A Westgard multirule combination was used to accept or reject runs [28]. The analytical imprecision comprised both the intra- and the interassay variation because each sample from an individual subject was analyzed consecutively in different assay runs.

### Criteria to interpret CA125 increments

**Figure 1.**
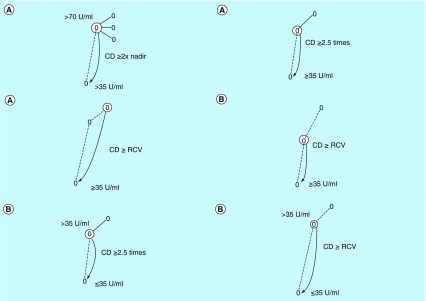
**Assessment criteria for serial CA125 concentrations during patient monitoring.** **(A)** CA125 increments starting above the cut-off. **(B)** CA125 increments starting below the cutoff. CD denotes the required critical difference. RCV denotes the reference change value. ^†^Criterion 1B showed the best monitoring performance for increments starting above the applied cut-off in the simulation models [23]. ^‡^Criterion 2A showed the best monitoring performance for increments starting below the applied cut-off in the simulation models [23].

Seven criteria were tested during first-line chemotherapy and the subsequent follow-up period (Figure 1). Four criteria were tested for increments starting from baseline concentrations above cut-off to higher levels (Figure 1A), and three criteria were tested for increments from below to above cut-off (Figure 1B). In both situations, Rustin *et al*. used an approach by which the increment between two concentrations had to exceed a defined arbitrary percentage of change before considered indicative of progression [6,8]. Tuxen *et al*. [20] used two approaches to generate their CA125 assessment criteria. Their first approach involved a statistical estimate of the significance of an increment based on the analytical and within-subject biological variations of CA125, termed the reference change value. Their second approach was similar to the approach used by Rustin *et al*. [6,8,22].

The assessment criteria were applied to the serial CA125 concentrations for each patient; the date of CA125 progression provided by each criterion was registered; and the clinical and marker evaluations in terms of progression were matched. The time interval between CA125 progression and clinical progression (lead time) for each criterion was calculated. The lead time was positive (>0 days) when marker progression preceded clinical progression. There was no lead time (= 0 days) if the clinical progression and the marker progression were obtained simultaneously. The lead time was negative (<0 days) when the clinical progression preceded the marker progression. When a clinical evaluation and a marker assessment differed, the data were registered as discordant; identical data were registered as concordant. Thus, the true-positive (TP) results denoted concordant information in terms of progressive disease with lead time ≥0 days. True-negative (TN) results denoted concordant information in terms of nonprogression. False-negative (FN) results denoted discordant information when there was clinical progression without CA125 progression. False-positive (FP) results denoted discordant information when CA125 progression was not followed by clinical progression.

### Statistics

A power calculation of the sample size was performed prior to opening the Phase II trial database. For a criterion to be valid for detecting CA125 increments it was assumed that the criterion should provide 70 TP signals in terms of progressive disease. By fixating the type 1 error on 0.05 and the type 2 error on 0.10, calculation of power showed that a total of 156 patients should be included for each criterion in order to detect a difference in their performance. The number of TP, FN, FP and TN results was counted. The sensitivities (the percentage of patients with tumor growth detected by CA125 increments), the specificities (the percentage of patients without new tumor growth confirmed by unchanged CA125 concentrations), the positive predictive values (the probability of clinical progression following CA125 progression), the negative predictive values (the probability of clinical nonprogression, given a marker nonprogression), the FP rates (the percentage of CA125 increments among patients without new tumor growth) and the FN rates (the percentage without CA125 increments among patients with new tumor growth) were calculated. The 95% CI were estimated according to Geigy formulas 771 and 772 [29].

## Results

A total of 231 patients were allocated to first-line chemotherapy. Forty-two patients were excluded: 16 due to insufficient sampling, 15 for other primary malignancy, 6 for death ≤4 weeks after initiation of therapy and 5 enrolled by mistake. When including all histological tumor types, 189 patients were eligible for CA125 monitoring during therapy and 143 patients were eligible for CA125 monitoring during the subsequent follow-up period. During therapy, 1385 specimens were collected with a median of seven samples per patient (range: 3–18 samples). The sampling interval ranged from 3 to 84 days. The CA125 monitoring period was in median 125 days (range: 21–390 days). During follow-up, 2214 specimens were collected with a median of ten samples per patient (range: 3–56 samples). The sampling interval ranged from 4 to 180 days. The CA125 monitoring period was in median 330 days (range: 45–2160 days). Characteristics of the 189 eligible patients at the start of the first-line chemotherapy appear in Table 1 and the treatments in Table 2. A total of 134 patients developed clinical progression cumulated from therapy and follow-up. When considering serous tumors, only 112 patients were eligible for CA125 monitoring and 84 patients developed clinical progression cumulated from therapy and follow-up.

**Table 1.** **Characteristics of ovarian cancer patients eligible for CA125 monitoring.**

**Patients characteristics**	**189 patients eligible for first-line chemotherapy**
Age (years)	
– Median	59
– Range	33–78
FIGO stage of disease, no.	
– IC	14
– IIA	2
– IIB	11
– IIC	13
– IIIA	12
– IIIB	18
– IIIC	89
– IV	29
– Not reported	1
WHO performance [30], no.	
– 0	118
– 1	54
– 2	11
– 3	3
– 4	0
– Not reported	3
Histological type, no.	
– Serosa	112
– Mucinous	5
– Clear cell	9
– Endometrioid	13
– Undifferentiated	27
– Mixed	10
– Borderlines, mixed mullerian tumor, mixed mesodermal tumor, Brenner tumor, and peritoneal serous adenocarcinoma	12
– Not reported	1
Histological grade	
– Poorly differentiated, no.	86
– Moderately differentiated	34
– Well differentiated	19
– Not reported	50
Residual tumor size after surgery before chemotherapy, no.	
0 cm	16
<1 cm	50
1–10 cm	65
>10 cm	55
– Not reported	3

FIGO: International Federation of Gynecology and Obstetrics..

**Table 2.** **First-line chemotherapy among 189 patients eligible for CA125 monitoring.**

**Treatment modalities**	**No. of patients**
Paclitaxel and carboplatin	89
Paclitaxel, carboplatin and gemcitabine	42
Paclitaxel and cisplatin	39
Cyclophosphamide and carboplatin	9
Treosulfan	7
Paclitaxel	1
Cyclophosphamide, doxorubicin and cisplatin	1
Carboplatin	1

### Accuracy of the applied CA125 assessment criteria

Based on data cumulated from first-line chemotherapy, the subsequent follow-up and all histological tumor types, the accuracies of the criteria to detect CA125 increments starting from above and below the applied cut-off were similar with overlapping 95% CI (Table 3A). Criteria 1A–1D did not provide FP increments but the numbers of FN events were high, 53–70%. Criteria 2A–2C provided 3–4 FP increments (same patients except one); all FP increments were registered during the post-therapy follow-up period at different time points depending on the individual criterion. The numbers of FN events were high (49–59%) (Table 3A). Inclusion of serous tumor types only, did not improve the accuracy (Table 3B). Thus, the number of FN events remained high both among patients with above and below cut-off concentrations (45–60 and 47–55%, respectively) (Table 3B). Overall, the positive predictive values of CA125 increments tended to be higher than the negative predictive values of stable concentrations.

**Table 3.** **Ability of the applied CA125 assessment criteria to identify and exclude progressive ovarian cancer cumulated from first-line chemotherapy and follow-up.**

**Criteria as provided in Figure 1**	**Number of TP, FP, FN and TN events**	**Sensitivity,% (95% CI)**	**Specificity,% (95% CI)**	**Positive predictive value, % (95% CI)**	**Negative predictive value, % (95% CI)**	**FP rate, % (95% CI)**	**FN rate, % (95% CI)**	**Ref.**
***3A All histological tumor types***								
Criterion 1A, Rustin *et al*.^†^	14 TP, 0 FP 16 FN, 8 TN	47 (31–63)	100 (67–100)	100 (79–100)	33 (18–52)	0 (0–33)	53 (37–6)	[8]
Criterion 1B, Tuxen *et al*.^†^	9 TP, 0 FP 21 FN, 8 TN	30 (17–47)	100 (67–100)	100 (70–100)	28 (15–45)	0 (0–33)	70 (53–83)	[7,20]
Criterion 1C, Tuxen *et al*.^†^	14 TP, 0 FP 16 FN, 8 TN	47 (31–63)	100 (67–100)	100 (79–100)	33 (18–52)	0 (0–33)	53 (37–69)	[7,20]
Criterion 1D, Tuxen *et al*.^†^	13 TP, 0 FP 17 FN, 8 TN	43 (28–60)	100 (67–100)	100 (78–100)	32 (17–51)	0 (0–33)	57 (40–72)	[7,20]
Criterion 2A, Rustin *et al*.^‡^	43 TP, 3 FP 61 FN, 44 TN	41 (33–50)	94 (84–98)	94 (84–98)	42 (39–51)	6 (2–16)	59 (50–67)	[6]
Criterion 2B, Tuxen *et al*.^‡^	50 TP, 3 FP 54 FN, 44 TN	48 (40–57)	94 (84–98)	94 (86–98)	45 (36–54)	6 (2–16)	52 (43–60)	[7,20]
Criterion 2C, Tuxen *et al*.^‡^	53 TP, 4 FP 51 FN, 43 TN	51 (43–59)	92 (81–97)	93 (84–98)	46 (37–55)	8 (3–19)	49 (41–57)	[7,20]
***3B Serous epithelial tumors***								
Criterion 1A, Rustin *et al*.^§^	9 TP, 0 FP 11 FN, 5 TN	45 (26–65)	100 (52–100)	100 (70–100)	31 (13–55)	0 (0–48)	55 (35–74)	[8]
Criterion 1B, Tuxen *et al*.^§^	8 TP, 0 FP 12 FN, 5 TN	40 (22–61)	100 (52–100)	100 (66–100)	29 (12–53)	0 (0–48)	60 (39–78)	[7,20]
Criterion 1C, Tuxen *et al*.^§^	11 TP, 0 FP 9 FN, 5 TN	55 (35–74)	100 (52–100)	100 (74–100)	36 (15–61)	0 (0–48)	45 (26–65)	[7,20]
Criterion 1D, Tuxen *et al*.^§^	10 TP, 0 FP 10 FN, 5 TN	50 (30–70)	100 (52–100)	100 (72–100)	33 (14–58)	0 (0–48)	50 (30–70)	[7,20]
Criterion 2A, Rustin *et al*.^¶^	29 TP, 3 FP 35 FN, 20 TN	45 (35–56)	87 (69–96)	91 (77–97)	36 (26–84)	13 (4–31)	55 (44–65)	[6]
Criterion 2B, Tuxen *et al*.^¶^	32 TP, 3 FP 32 FN, 20 TN	50 (39–61)	87 (69–96)	91 (79–98)	39 (27–51)	13 (4–31)	50 (39–61)	[7,20]
Criterion 2C, Tuxen *et al*.^¶^	34 TP, 4 FP 30 FN, 19 TN	53 (42–64)	83 (64–94)	90 (77–96)	39 (27–52)	17 (6–36)	47 (36–58)	[7,20]

TP denotes true-positive results: concordant clinical and CA125 progression with a lead time of ≥0 days.

FP denotes false-positive results: discordant clinical and CA125 information with CA125 progression without clinical progression.

TN denotes true-negative results: concordant clinical and CA125 information in terms of nonprogression.

FN denotes false-negative results: discordant clinical and CA125 information with clinical progression without CA125 progression or negative CA125 lead time.

( ): Two-sided 95% CI (%).

^†^CA125 baseline concentration above the applied cut-off among 38/189 eligible patients; 30/38 patients developed clinical progression.

^‡^CA125 baseline concentration below the applied cut-off among 151/189 eligible patients; 104/151 patients developed clinical progression.

^§^CA125 baseline concentration above the applied cut-off among 25/112 eligible patients; 20/25 patients developed clinical progression.

^¶^CA125 baseline concentration below the applied cut-off among 87/112 eligible patients; 64/87 patients developed clinical progression.

### CA125 lead times

#### All histological tumor types

About 20% (38/189) of the eligible patients had CA125 baseline concentrations above cut-off and 79% (30/38) of the patients developed clinical progression (Table 4A). Depending on the criterion, 30–47% of the patients presented with lead times ≥0 days (median: 27–87 days). The percentage of patients with lead times >0 days and ≤0 days ranged from 23–43 to 3–10%, respectively. About 80% (151/189) of the eligible patients had CA125 baseline concentrations below cut-off and 69% (104/151) of the patients developed clinical progression. Depending on the criterion, 41–51% of the patients presented with lead times ≥0 days (median: 41–46 days). The percentage of patients presenting with lead times >0 days and ≤0 days ranged from 34–49 to 14–29%, respectively.

**Table 4.** **CA125 lead times among progressive ovarian cancer patients provided by the applied assessment criteria cumulated from first-line chemotherapy and follow-up.**

**Criteria as provided in Figure 1**	**Median, days (range) No. (%)**	**≥0 days No. (%)**	**>0 days No. (%)**	**≤0 days No. (%)**	**= 0 days No. (%)**	**<0 days No. (%)**	**Ref.**
***Lead times***							
***4A All histological tumor types***							
Criterion 1A, Rustin *et al*.^†^	60 (0–248)	14 (47)	13 (43)	1 (3)	1 (3)	0	[8]
Criterion 1B, Tuxen *et al*.^†^	27 (0–257)	9 (30)	7 (23)	3 (10)	2 (7)	1 (3)	[7,20]
Criterion 1C, Tuxen *et al*.^†^	87 (0–356)	14 (47)	13 (43)	2 (7)	1 (3)	1 (3)	[7,20]
Criterion 1D, Tuxen *et al*.^†^	66 (0–356)	13 (43)	12 (40)	3 (10)	1 (3)	2 (7)	[7,20]
Criterion 2A, Rustin *et al*.^‡^	41 (0–369)	43 (41)	35 (34)	30 (29)	8 (8)	22 (21)	[6]
Criterion 2B, Tuxen *et al*.^‡^	45 (0–775)	50 (48)	48 (46)	15 (14)	2 (2)	13 (13)	[7,20]
Criterion 2C, Tuxen *et al*.^‡^	46 (0–775)	53 (51)	51 (49)	16 (15)	2 (2)	14 (13)	[7,20]
***4B Serous epithelial tumors***							
Criterion 1A, Rustin *et al*.^§^	66 (0–248)	9 (45)	8 (40)	1 (5)	1 (5)	0	[8]
Criterion 1B, Tuxen *et al*.^§^	26 (0–75)	8 (40)	6 (30)	2 (10)	2 (10)	0	[7,20]
Criterion 1C, Tuxen *et al*.^§^	85 (0–169)	11 (55)	10 (50)	1 (5)	1 (5)	0	[7,20]
Criterion 1D, Tuxen *et al*.^§^	76 (0–169)	10 (50)	9 (45)	2 (10)	1 (5)	1 (5)	[7,20]
Criterion 2A, Rustin *et al*.^¶^	30 (0–269)	29 (45)	25 (39)	18 (28)	4 (6)	14 (22)	[6]
Criterion 2B, Tuxen *et al*.^¶^	47 (0–406)	32 (50)	31 (48)	7 (11)	1 (2)	6 (9)	[7,20]
Criterion 2C, Tuxen *et al*.^¶^	52 (0–406)	34 (53)	33 (52)	9 (14)	1 (2)	8 (13)	[7,20]

^†^Criteria 1A–1D assessed CA125 increments among 20% (38/189) of the eligible patients with baseline concentrations above the applied cut-off; 79% (30/38) of the patients developed clinical progression.

^‡^Criteria 2A–2C to assess CA125 increments among 80% (151/189) of the eligible patients with baseline concentrations below the applied cut-off; 69% (104/151) of the patients developed clinical progression.

^§^Criteria 1A–1D assess CA125 increments among 22% (25/112) of the eligible patients with baseline concentrations above the applied cut-off; 80% (20/25) of the patients developed clinical progression.

^¶^Criteria 2A–2C to assess CA125 increments among 78% (87/112) of the eligible patients with baseline concentrations below the applied cut-off; 74% (64/87) of the patients developed clinical progression.

#### Serous tumors

**Figure 2.**
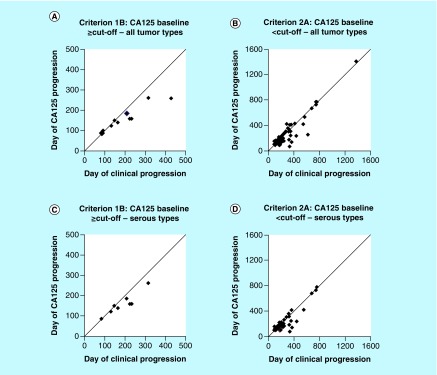
**Distribution of CA125 lead times from individual patients cumulated from first-line chemotherapy and postchemotherapy follow-up.** **(A)** CA125 lead time provided by criterion 1B among ten individual patients with clinical progression. **(B)** CA125 lead time provided by criterion 2A among 65 individual patients with clinical progression. **(C)** CA125 lead time provided by criterion 1B among eight individual patients with clinical progression. **(D)** CA125 lead time provided by criterion 2A among 43 individual patients with clinical progression. ^†^Each dot represents the CA125 lead time obtained from a single patient. The dots below the solid line represent the individual positive lead time (>0 days) where CA125 progression preceded clinical progression. The dots on the solid line represent events where the individual lead time was zero (= 0 days) because the date of clinical progression coincided with the date of CA125 progression. The dots above the solid line represent the individual negative lead time (<0 days) where clinical progression preceded CA125 progression.

About 22% (25/112) of the eligible patients had CA125 baseline concentrations above cut-off and 80% (20/25) of the patients developed clinical progression (Table 4B). Depending on the criterion, 40–55% of the patients presented with lead times ≥0 days (median: 26–85 days). The percentage of patients with CA125 lead times >0 days and ≤0 days ranged from 30–50 to 5–10%, respectively. About 78% (87/112) of the eligible patients had CA125 baseline concentrations below cut-off and 74% (64/87) of the patients developed clinical progression. Depending on the criterion, 45–53% of the patients presented with lead times ≥0 days (median: 30–52 days). The percentage of patients presenting with lead times >0 days and ≤0 days ranged from 39–52 and 11–28%, respectively. Overall, the lead times observed among all histological tumor types and serous tumors only appeared similar (Table 4A & B). Figure 2 illustrates a new format to present detailed information of lead times in terms of progression among individual patients. Events with positive lead times (>0 days) are marked below the solid line; events with no lead time (=0 days) are marked on the solid line; and events with negative lead times (<0 days) are marked above the solid line.

## Discussion

Tumor marker monitoring studies investigating the accuracy of criteria to detect increasing concentrations have frequently been based on heterogeneous designs making interpretation of results difficult [31]. The current study followed the proposals of the EGTM and was designed as a prospective Phase II biomarker monitoring trial embedded into clinical drug trials where the tumor marker investigation was a secondary objective in relation to the clinical drug trial [26]. The current Phase II monitoring trial investigated the best performing criteria previously identified in a Phase I biomarker simulation study. The criteria were investigated among a cohort of ovarian cancer patients with disease stages IC–IV receiving first-line chemotherapy and during the subsequent post-therapy follow-up period. A distinction was made when interpreting CA125 increments starting from baseline concentrations above and below cut-off, respectively, because published criteria are focused on the nadir concentration of the increment in relation to the applied cut-off concentration.

It appears from Table 3A & B that approximately 80% of the patients had baseline CA125 concentrations below the applied cut-off. This was due to the inclusion procedure where 21% of the patients had early-stage disease (FIGO IA–IIC) with a low tumor burden (Table 1). For increments starting from concentrations above cut-off, neither of the criteria 1A–1D provided FP signals when all histological ovarian tumor types were considered (Table 3A). For baseline concentrations starting below cut-off, criteria 2A–2C each provided one temporary FP increment to above cut-off at different time points among the same three patients during post-therapy follow-up (Table 3A). Criterion 2C provided an additional asynchronous FP increment. Three patients were under surveillance for additional 2–4 years without developing clinical progression, and one patient was followed for 6 months before the 5-year routine surveillance was completed and the patient was discharged from further follow-up without evidence of disease. This condition illustrates one of the most difficult situations in monitoring where patients have a rising CA125, no evidence of progressive disease on imaging and no clinical symptoms of disease progression. It may be suggested that the patient should have been offered an additional year of surveillance. Bias may be less likely as the cause of the observed FP events because all measurements were performed at the same laboratory closely following the analytical quality. Additionally, the FP increments did not occur at the time point when the method to measure CA125 was changed. However, it may be speculated that undetected temporary benign disease may have caused the FP increments [22,32].

Criteria 1A and 2A were developed to monitor patients during follow-up after primary therapy and have not been validated during first-line chemotherapy; consequently, it is difficult to compare the current data with former studies. As regards criteria 1B–1D and 2B–2C, this is the first clinical study to report on their individual accuracy; their combined accuracy has been reported [7,20]. Overall, the accuracies of the criteria to interpret increments from above cut-off to higher levels among all histological tumor types were similar as were the accuracies of the criteria interpreting increments from below to above cut-off (Table 3A). However, a closer examination indicates that the accuracies of the criteria in the current Phase II monitoring trial may support the accuracies reported in a previous Phase I simulation study [23]. Based on simulated data stratified for the lowest number of FP increments criterion 1B performed best among increments starting from above cut-off followed by criteria 1A, 1D and 1C, respectively; and criterion 2A performed best among increments starting from below cut-off followed by criteria 2B and 2C. Owing to the nature of quantitative biochemical tests, there is an inverse relationship between the number of FP and FN events; consequently, the lower the number of FP events, the higher the number of FN events [33]. Accordingly, the criteria with the lowest number of FP events in the Phase I simulation tests (criteria 1B and 2A) provided the highest number of FN events in the current Phase II monitoring trial (Table 3A).

It is often stated that CA125 is mainly expressed by ovarian tumors of the serous type. We therefore investigated the accuracy among patients whose tumors had this histological classification (Table 3B). However, the accuracy among serous tumors did not improve as compared with the accuracy when all histological types were included (Table 3B & A, respectively). In both situations, the observed numbers of FN events were relatively high indicating that the validated CA125 criteria are unreliable to exclude clinical progression independent of the baseline concentrations. Again, the criteria with the lowest number of FP events in the Phase I simulation tests (criteria 1B and 2A) provided the highest number of FN events in the current Phase II monitoring trial (Table 3B). Other issues should also be considered in association with the reported FN events. Since the study period 1995–2001, the histological classification system has been changed. Recent evidence has identified EOC as a heterogeneous disease with five distinct subtypes: high-grade serous, low-grade serous, clear cell, endometrioid and mucinous. Each subtype is associated with different biological characteristics, clinical behavior and prognosis [34,35]. There is now persuasive evidence to classify these five types of ovarian carcinoma as different diseases [34,36,37]. Reclassification of the patients according to current standards may provide an alternative distribution in the different subtypes. Situations with slow rate of CA125 increase due to low production in the tumor represent a challenge in terms of FN events. In an effort to elucidate whether the rate of FN events can be reduced without numerous FP signals, it seems relevant to validate criteria specially designed to assess increments within the normal range and from below to slightly above the applied cut-off [38]. The reliability and the length of the positive lead time are important parameters for monitoring of cancer patients. A positive lead time enables early supplementary investigations, in other words, imaging and/or institution/change of therapy. The lead time provided by the criteria during clinical monitoring was in accordance with the lead-time potential obtained in the simulation studies. The criteria with the longest lead times in the current clinical Phase II monitoring trial provided the shortest time interval needed to detect 100% of TP CA125 increments in the simulation study [23]. A multicenter study by Rustin *et al*. reported on women with histologically confirmed epithelial ovarian, fallopian tube or serous primary peritoneal cancer in complete remission after first-line chemotherapy and baseline CA125 concentrations <35 U/ml [24]. When CA125 concentrations rose to ≥70 U/ml during the follow-up, the patients were randomized to early second-line chemotherapy or initiation of therapy at clinical or symptomatic relapse. They demonstrated a median lead time of 4.8 months; however, early treatment based on CA125 increments led to more chemotherapy, no difference in survival and worse quality of life. The median lead time in the current study among patients with CA125 increments starting from baseline CA125 concentrations <35 U/ml cumulated from first-line chemotherapy and follow-up was 1.0–1.7 months (Table 4A & B). It is difficult to argue that the short lead times observed in the current investigation would benefit the patients in terms of prolonged survival following early CA125-guided therapy.

One of the limitations of the current study is that the investigation was not blinded; the CA125 data were available throughout the study period together with the results of clinical examinations and imaging [25]. This may have influenced the length of the positive lead time because the clinicians had the opportunity to request earlier imaging based on CA125 increments and thereby shorten the potential lead time. Another weakness is the changed histological classification of EOC which now considered a heterogeneous disease with five different subtypes [35]. Most likely, the new classification system will provide a different distribution of patients among the two groups, all tumors and serous tumors only, respectively, inflicting the presented results with some uncertainty.

A further weakness adheres to the clinical response evaluation, which was based on criteria of the WHO in use at the time of the present study [27]. In 2000, the WHO standards were replaced by a set of new guidelines to evaluate the RECIST [39]. Re-evaluation of clinical response among the investigated patients according to the new standards may have some impact on the obtained results, but would hardly influence the overall impression of the validity of CA125 as a monitor of patients with EOC. Further limitations of the study could be due to the fact that first-line chemotherapy and the subsequent follow-up period were not investigated individually because there were not enough patients entering the follow-up period allowing a meaningful statistical analysis, and the performance of each criterion was not investigated individually for each stage of disease due to a low number of patients within the individual subgroups.

Overall, the study supports a previous multicenter investigation suggesting that CA125 information cannot stand alone but should be used in conjunction with other investigative procedures [24]. Performing scheduled CA125 testing to follow patients has a significant cost. Therefore, it should be considered whether sustaining this cost is relevant without substantial benefit for the individual patient. In conclusion, the applied CA125 assessment criteria showed low sensitivity (30–55%), low negative predictive value (28–46%), high positive predictive value (90–100%) and short median lead time (26–87 days) among several patients.

## Conclusion & future perspective

There is a need for supplementary markers and alternative assessment criteria for patient surveillance.

The monitoring performance of the promising biomarker HE4 in combination with CA125 needs further investigated among EOC patients undergoing first-line chemotherapy and during the subsequent follow-up period.

Evidently, identification of new biomarkers is important, and the area is developing fast, in other words, circulating tumors cells, DNA and RNA fragments as well as epigenetic alterations [40,41].

Guidelines for conducting monitoring studies provided by the EGTM may be helpful when designing investigations of new serological markers for ovarian cancer [26,42].

**Summary points**

**Background**

- The current Phase II study investigated the performance of seven CA125 criteria to monitor progressive ovarian cancer.

**Material & method**

- Four criteria were used to asses CA125 increments starting from concentrations ≥35 U/ml and three criteria to asses increments starting from concentrations <35 U/ml.

**Results**

- The performances of the CA125 criteria were similar with sensitivities of 30–55%, negative predictive values of 28–46%, positive predictive values of 90–100% and median lead times of 26–87 days.

**Discussion**

- The current study supports a previous multicenter investigation suggesting that CA125 information cannot stand alone but should be used in conjunction with other investigative procedures.
